# Range extension and conservation status of the rare Solanaceae shrub, *Solanumconocarpum*

**DOI:** 10.3897/BDJ.9.e69156

**Published:** 2021-07-23

**Authors:** Sara Barrios, Omar A. Monsegur-Rivera, Thomas M Heller, Natasha Harrigan, Keith A Grant, Eleanor Gibney, Colin P. Clubbe, Martin A. Hamilton

**Affiliations:** 1 Royal Botanic Gardens, Kew, Richmond, United Kingdom Royal Botanic Gardens, Kew Richmond United Kingdom; 2 US Fish and Wildlife Service, Boquerón, Puerto Rico US Fish and Wildlife Service Boquerón Puerto Rico; 3 National Parks Trust of the Virgin Islands, Road Town, Virgin Islands (British) National Parks Trust of the Virgin Islands Road Town Virgin Islands (British); 4 Independent Researcher, Saint John, Virgin Islands (USA) Independent Researcher Saint John Virgin Islands (USA); 5 Oak Spring Garden Foundation, Upperville, VA, United States of America Oak Spring Garden Foundation Upperville, VA United States of America

**Keywords:** Caribbean flora, conservation status, distribution, endemism, new records, Red List, Solanaceae

## Abstract

**Background:**

The British Virgin Islands and the US Virgin Islands, two island groups located in the Caribbean archipelago, hold unique plant diversity and high endemism. Until recently, *Solanumconocarpum* was considered a rare plant species endemic to the island of St. John in the US Virgin Islands. Ongoing botanical surveys in this region are revealing new populations and refining our understanding of the distribution of these narrow endemic plant species. The objective of this paper is to assess the conservation status of *S.conocarpum*, including a review of its geographic range, population numbers, threats and conservation actions needed for its long-term survival.

**New information:**

In this paper, we present new occurrences for *S.conocarpum*, extending its geographic range to a new island, Tortola and new territory, the British Virgin Islands. Despite this range expansion, this species is evaluated as Endangered (EN), based on Criteria B1b(iii,v)+2b(iii,v)+C2a(i), according to the IUCN Red List Categories and Criteria. The extent of occurrence (EOO = 46 km^2^) and area of occupancy (AOO = 20 km^2^) are highly restricted. On St. John (US Virgin Islands), the historically recorded individuals at Reef Bay, Europa Ridge and Sabbat Point are now considered extirpated due to disturbance from development compounded by invasive species, as well as the impact of feral ungulates and drought stress. These threats are impacting the species across the whole island of St. John and contributing to a continuing decline of suitable habitat, despite the island being a National Park. On the island of Tortola, the species occurs on unprotected lands subject to development and habitat modification and decline by feral ungulates. Based on these threats acting separately across the two islands, two locations were defined. The estimated total number of mature individuals ranges between 150 and 250, with the largest subpopulation at Nanny Point in the US Virgin Islands, containing 108 mature individuals. Conservation action, focused on protecting this species' habitat, is urgently needed.

## Introduction

The Caribbean archipelago is a biodiversity hotspot with high levels of endemism and threatened taxa (Myers et al. 2000). The British Virgin Islands (BVI) and the neighbouring US Virgin Islands (USVI) are a group of Caribbean islands which share many botanical similarities, including several endemic plant species (Acevedo-Rodriguez 1996, Heller 2019).

**Figure 1.**
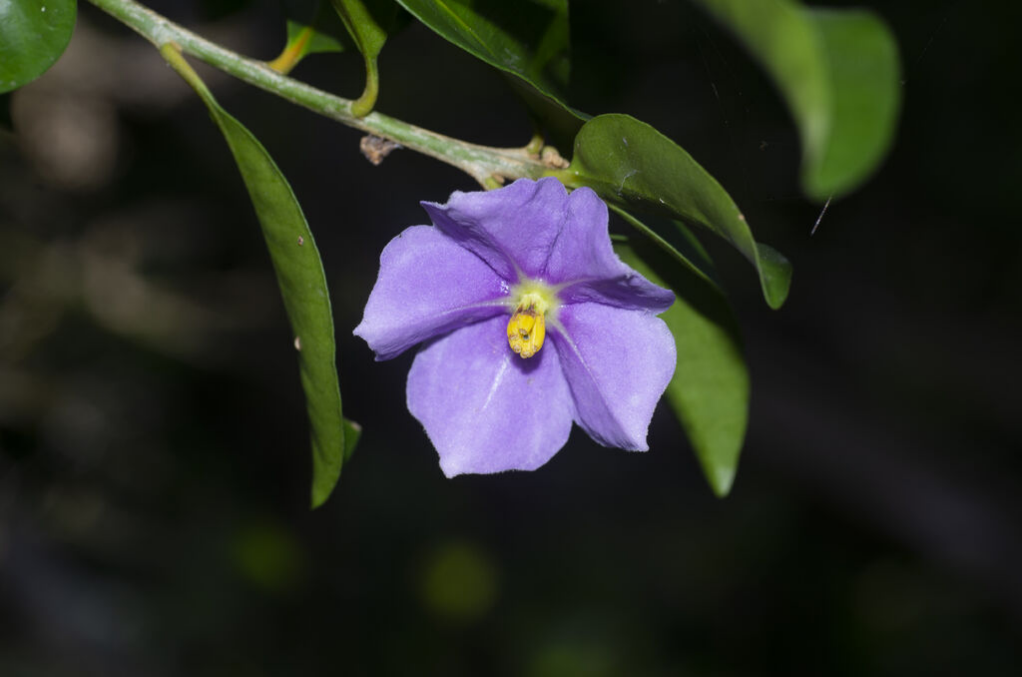
Flower of *Solanumconocarpum* on recently discovered plants on the island of Tortola, BVI. Image by Thomas Heller.

**Figure 2.**
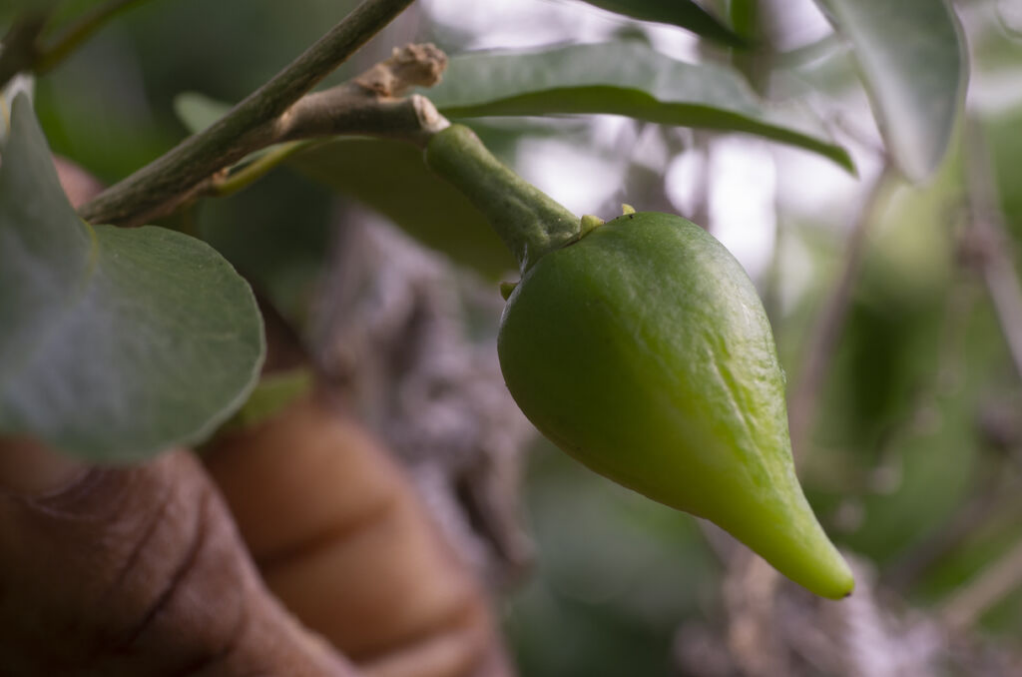
Developing fruit on a recently discovered plant of *Solanumconocarpum* on the island of Tortola, BVI. Image by Thomas Heller.

*Solanumconocarpum* Dunal was previously thought to be endemic to the island of St. John in the USVI (Acevedo-Rodriguez 1996). The species was described by Dunal in 1813, based on material collected by French botanist, Louis M. Richard at Coral Bay, St. John in 1787. The species was believed to be extinct as it remained uncollected for over a century until it was rediscovered by Pedro Acevedo-Rodríguez as part of his work on the Flora of St. John (Acevedo-Rodriguez 1996, U.S. Fish and Wildlife Service 2019). In 2002, a new subpopulation was discovered at Nanny Point on St. John, revealing many more individuals than initially thought (U.S. Fish and Wildlife Service 2019). In June 2018, a juvenile plant with vegetative characters matching the species was collected (M.A. Hamilton, #1758, DNA Silica Bank S3977, K!) during a botanical survey at Sabbath Hill on the island of Tortola (BVI). Later that year, a targeted survey to the same location was unable to locate the original plant; however, further exploration in the surrounding forest revealed several plants of *S.conocarpum*, including flowering (Fig. 1) and fruiting (Fig. 2) individuals. Vouchers collected (N. Harrigan, #155, K000817185, K!; #156, K000817186, K!) confirmed the range expansion of this species to the island of Tortola (Heller et al. 2018). Despite belonging to different jurisdictions, the islands of Tortola (British Virgin Islands) and St. John (US Virgin Islands) are separated by less than 2 km of water at their nearest points.

**Figure 3.**
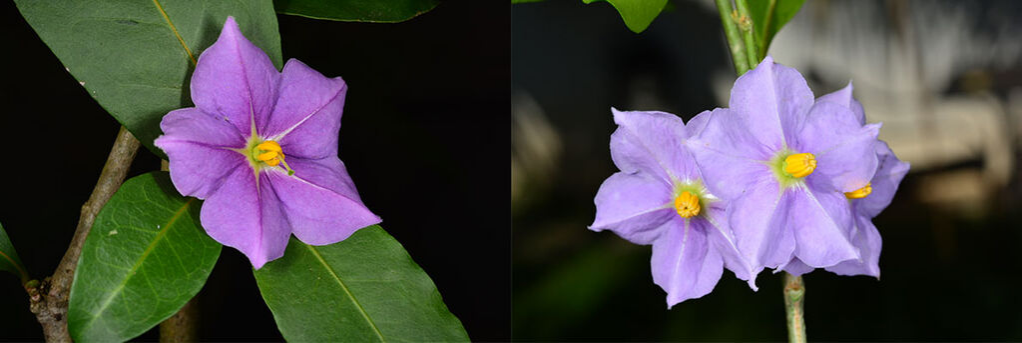
Long-styled (left) and short-styled flowers (right) of *Solanumconocarpum*. Images by Omar Monsegur.

**Figure 4.**
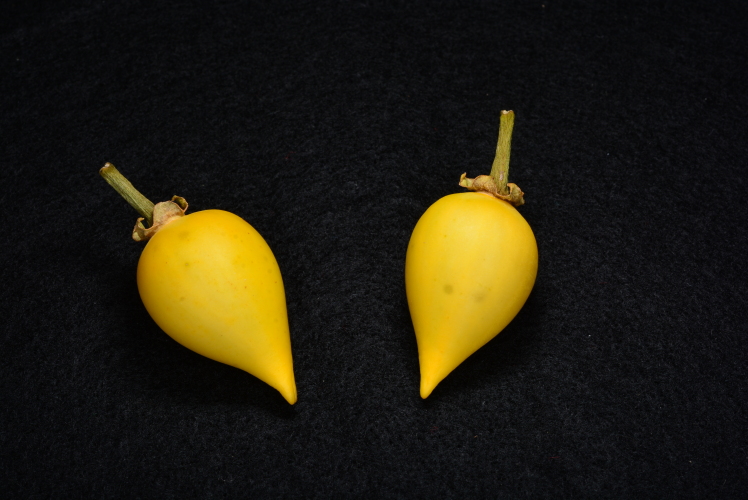
Ripe yellow fruit of *Solanumconocarpum*. Image by Omar Monsegur.

*Solanumconocarpum* is a small shrub which can grow up to 3 metres tall in the wild. The leaves are oblong-elliptic or oblanceolate, ranging in size from 3.5 to 7.0 cm long and 1.6 to 3.0 cm wide, coriaceous, glabrous, except for new growth and with a conspicuous orange to yellow midvein. The flowers are heterostylous (Fig. 3), growing in nearly sessile lateral or terminal cymes. The corolla is about 2 cm wide with five violet coloured petals. The fruit is a berry, ovoid conical in shape, green when immature and turning yellow when ripe (Fig. 4) (Acevedo-Rodriguez 1996, U.S. Fish and Wildlife Service 2019, PBI Solanum Project 2020). *Solanumconocarpum* occurs in dry deciduous and coastal forests with a varied range of associated woody species that lack a characteristic species composition. The species usually grows as an understorey plant responding favourably to disturbance (Stanford et al. 2013, Lindsay et al. 2015, Heller et al. 2018). A habitat suitability model for this species, for the island of St. John, identified 695 hectares of high-quality habitat, 1,275 hectares of moderate-quality habitat, 2,912 hectares of low-quality and poor-quality habitat and 187 hectares of unsuitable habitat (Palumbo et al. 2016). Some of these areas have already been surveyed but a full validation of this model is needed (Monsegur, pers. obs. 2021).

Previous studies by Stanford et al. (2013) suggested that individuals of this species are self-incompatible, possiby due to stigmatic differences between flowers, rather than genetic-based self-incompatibility. However, recent observations of isolated, cultivated material have shown certain individuals to be self-compatible, producing copious fruits, bearing viable seeds and producing abundant seedlings below the mother plant (U.S. Fish and Wildlife Service 2019). The lack of evidence of natural recruitment cannot be attributed to low seed viability as germination under greenhouse conditions has been recorded at almost 100% (Stanford et al. 2013). Under greenhouse conditions, the species can reach a reproductive size between 7 to 16 months from germination (Anderson et al. 2015; Gibney, pers. obs. 2021); however, this period is expected to be greater in the wild due to environmental stochasticity. In addition, exotic mammals are probably limiting seedling establishment by directly grazing on young plants and modifying the structure of the vegetation, which may also result in changes in microhabitat conditions that may affect seed germination and seedling recruitment of this species (Island Conservation 2018, U.S. Fish and Wildlife Service 2019).

**Figure 5.**
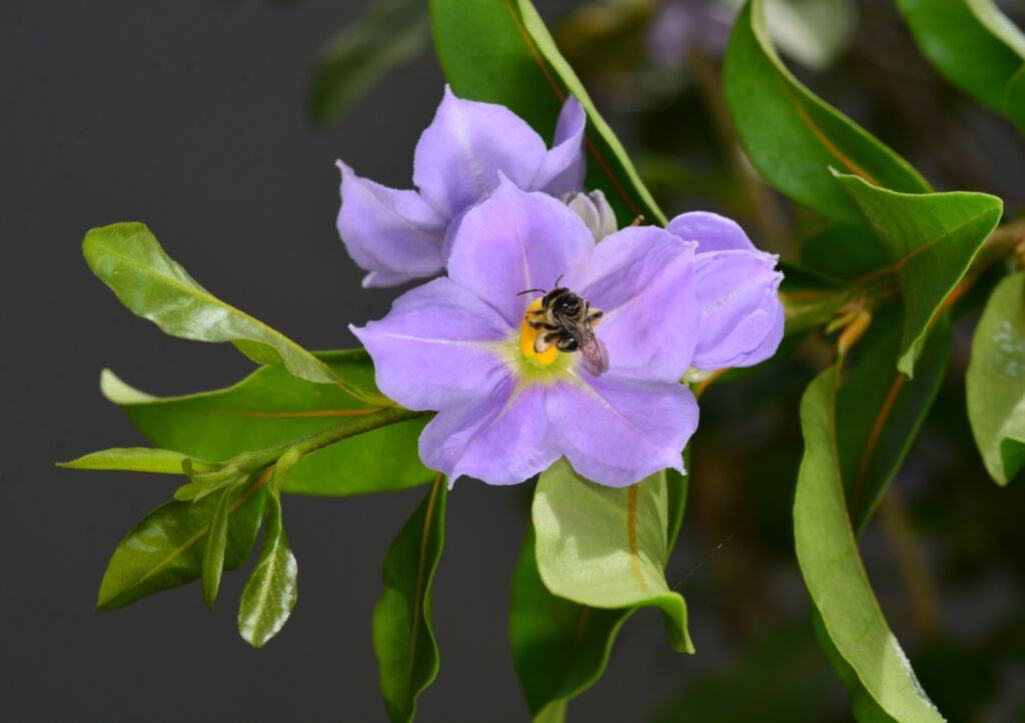
*Solanumconocarpum* flower being visited by *Exomalopsisbahamica*, a bee native to the Caribbean. Image by Omar Monsegur.

Regarding possible pollinators for this species, there are reports of *Xylocopamordax* Smith, 1874 and *Apismellifera* Linnaeus, 1758 actively visiting the flowers. These observations were followed by a massive fruit production event, suggesting these insects may be effective pollinators for *S.conocarpum*. Seed disperser vectors for this species remain unknown (U.S. Fish and Wildlife Service 2017, U.S. Fish and Wildlife Service 2019). Recently, *Exomalopsisbahamica* Timberlake, 1980, a native Caribbean bee, has been observed visiting cultivated material on Puerto Rico (Fig. 5) (Monsegur, pers. obs. 2021). Further research is needed to determine the reproductive biology and confirm the field observations.

## *Ex situ* conservation

**Figure 6.**
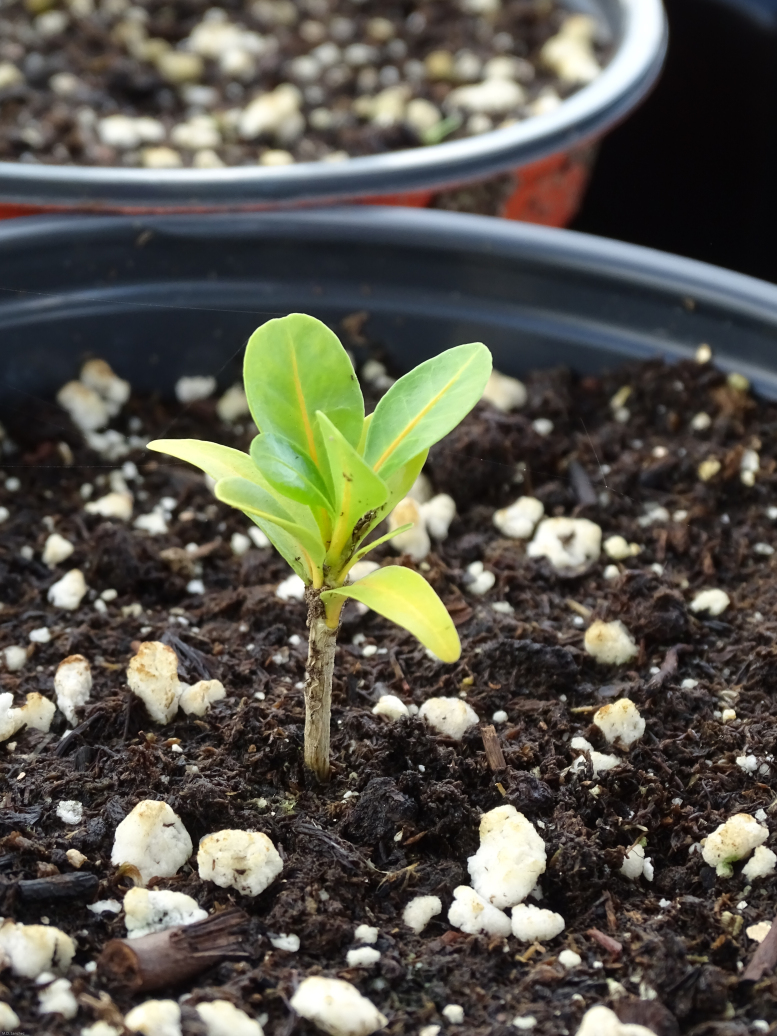
Recently propagated sapling of *Solanumconocarpum* grown from the seeds wild-collected at the J.R. O'Neal Botanic Gardens on Tortola, BVI. Image by Michele Dani Sanchez.

**Figure 7.**
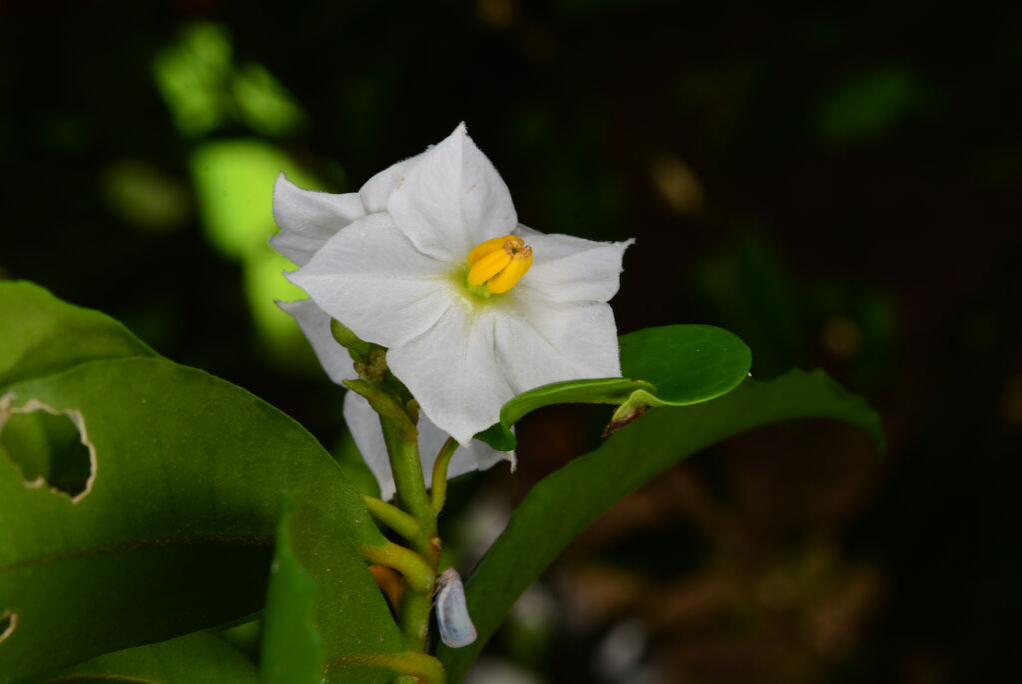
*Ex situ* collection of *Solanumconocarpum* displaying a white flower. Image by Omar Monsegur.

There are regional *ex-situ* collections established on the island of St. John and at St. Georges Village Botanical Garden on the island of St. Croix, both in the USVI and at the J.R. O’Neal Botanic Gardens on the island of Tortola in the BVI (Hamilton et al. 2018, Heller et al. 2018, Island Conservation 2018). Additionally, the species is under cultivation at the Fairchild Tropical Botanical Garden in Miami, USA. The majority of these collections are of material collected and propagated from the island of St. John (Gibney, pers. obs. 2021). The material at the J.R. O’Neal Botanic Gardens was propagated by cuttings and seed germination. Cuttings were taken from plants originally from St. John (Gibney, pers. obs. 2021) and from wild plants from the recently discovered site at Sabbath Hill on Tortola. These cuttings were propagated in perlite with a powder rooting hormone treatment. Once rooted, the cuttings were transferred to a commercial potting media. Seeds were also collected from the population located in Sabbath Hill on Tortola and sown in sterilised commercial potting media without any seed threatment and good germination success was recorded (Fig. 6). Flowers from cultivated plants seem to display a wider variation in size and colour than plants found in the wild. Plants cultivated in a private collection on St. John displayed flowers ranging from white (Fig. 7) to purple (Gibney, pers. obs. 2021). However, white-flowered plants have never been observed in wild populations.

In the USVI, the US Fish and Wildlife Service is working with the National Park Service (NPS) and local partners, including Friends of the Virgin Islands National Park, on the propagation, population enhancement and species reintroduction on St. John. Additionally, the US Fish and Wildlife Service is also collaborating with Island Conservation (IC) on research to address the role of feral ungulates and invasive plant species on the lack of natural *S.conocarpum* recruitment (Island Conservation 2018).

## Common name

The common name of *S.conocarpum* is "Marron Bacora" (Acevedo-Rodriguez 1996). "Marron" from the French language means “wild”, a term originally used to describe escaped enslaved people who were living in the wild. In the Taíno language, an Arawakan language spoken by the Taíno people who lived in the Caribbean, there is a similar word “Simaran” and, in Spanish, an equivalent term "Cimarron", both with a similar meaning. During the 18^th^ and 19^th^ centuries, the common name for sweet bananas used in the Virgin Islands was "Bacova". Many people detect a strong banana-like fragrance to the ripe fruit of *S.conocarpum*, which was presumably consumed by humans, like the other woody native *Solanum* species. It seems possible that the single-letter difference was a transcription error or a slight variation in pronunciation (Gibney, pers. obs. 2021).

## Methods

To gather information for the following conservation assessment, collections from the K, NY and US herbaria and many literature records have been digitised. Field observations contributed additional information on distribution, preferred habitat, threats and population sizes.

## Species Conservation Profiles

### Solanum conocarpum

#### Species information

Scientific name: Solanumconocarpum

Species authority: Dunal

Common names: Marron Bacora

Kingdom: Plantae

Phylum: Tracheophyta

Class: Magnoliopsida

Order: Solanales

Family: Solanaceae

Region for assessment: Global

#### Editor & Reviewers

##### Reviewers

Reviewers: Canteiro, C.

##### Editor

Editor: Barrios, S.; Monsegur, O.A.; Heller, T.M.; Harrigan, N.; Grant, K.A.; Gibney, E.; Clubbe, C.P.; Hamilton, M.A.

#### Reviewers

Reviewers: Canteiro, C.

#### Editor

Editor: Barrios, S.; Monsegur, O.A.; Heller, T.M.; Harrigan, N.; Grant, K.A.; Gibney, E.; Clubbe, C.P.; Hamilton, M.A.

#### Geographic range

Biogeographic realm: Neotropical

Countries: Virgin Islands, BritishVirgin Islands, U.S.

Map of records (Google Earth): Suppl. material 1

Basis of EOO and AOO: Observed

Basis (narrative): The extent of occurrence (EOO) was calculated to be 46 km^2^ and the area of occupancy to be 20 km^2^ based on a 2 × 2 km cell size, using GeoCAT (Bachman et al. 2011).

Min Elevation/Depth (m): 10

Max Elevation/Depth (m): 200

Range description: *Solanumconocarpum* is endemic to the British (BVI) and the US Virgin Islands (USVI). Previously thought to be restricted to the island of St. John in the USVI (Acevedo-Rodriguez and Strong 2012, Lindsay et al. 2015), this species was discovered on the island of Tortola in the BVI in 2018 and its identity was confirmed through further surveys and voucher collections (Hamilton et al. 2018, Heller et al. 2018). On St. John in the USVI, the species is known to be extant at five localities: Nanny Point, John's Folly, Brown Bay Trail, Brown Bay Ridge and Reef Bay Trail (U.S. Fish and Wildlife Service 2019). On Tortola in the BVI, this species is only known from Sabbath Hill (Hamilton et al. 2018, Heller et al. 2018). A sterile herbarium collection from Gorda Peak on Virgin Gorda in the BVI (E.L. Little, #23836, NY01284578, Image!; US00732108, Image!) was thought to be a record of this species; however, the material has recently been identified as another Solanaceae species, *Cestrumlaurifolium* L'Hér. (Knapp, pers. comm. 2020)

#### New occurrences

#### Extent of occurrence

EOO (km2): 46

Trend: Unknown

Causes ceased?: Unknown

Causes understood?: Unknown

Causes reversible?: Unknown

Extreme fluctuations?: No

#### Area of occupancy

Trend: Decline (observed)

Justification for trend: Despite the new subpopulation found on the island of Tortola in the BVI, three previously known subpopulations on the island of St. John in the USVI are now extirpated.

Causes ceased?: No

Causes understood?: Yes

Causes reversible?: Unknown

Extreme fluctuations?: No

AOO (km2): 20

#### Locations

Number of locations: Two

Justification for number of locations: The number of locations was calculated to be two considering feral animals are the main threat for the survival of this species through grazing either directly of individuals or indirectly causing habitat degradation. These threats are acting separately across the two islands.

Trend: Unknown

Extreme fluctuations?: No

#### Population

Number of individuals: 150-250

Trend: Decline (observed)

Justification for trend: Historical specimens collected at Reef Bay, Europa Ridge and Sabbat Point on St. John in the USVI are now considered as extirpated localities (U.S. Fish and Wildlife Service 2019). At Sabbat Point, a population recorded as extirpated by 2017 due to disturbance from development compounded by invasive species. In the case of the former populations along the south coast of St. John (e.g. Reef Bay Valley and Brown Bay Ridge), the main threats were feral ungulates and drought stress. Additionally, data gathered prior to the impacts of Hurricanes Irma and Maria in 2017 indicate that known subpopulations, such as Nanny Point, were declining (U.S. Fish and Wildlife Service 2019).

Causes ceased?: Unknown

Causes understood?: Unknown

Causes reversible?: Unknown

Extreme fluctuations?: No

Population Information (Narrative): This species is considered rare (Acevedo-Rodriguez 1996, Lindsay et al. 2015). The U.S. Fish and Wildlife Service (2017) found on the island of St. John an old population (Nanny Point) with predominantly mature individuals (large stem diameter) and lacking natural recruitment. It is estimated that plants exist, including mature individuals, juveniles and seedlings, at eight distinct localities. The largest number of individuals occurs at Nanny Point in the USVI, where approximately 108 mature individuals, 53 juveniles and 40 seedlings were recorded during population assessments following Hurricanes Irma and Maria (Island Conservation 2018, U.S. Fish and Wildlife Service 2019).The latest records from St. John include new localities like the Reef Bay Trail, where seven plants have been recorded and Brown Bay Ridge (U.S. Fish and Wildlife Service 2017, U.S. Fish and Wildlife Service 2019). In the BVI, 40 to 50 plants are known from Sabbath Hill (Heller et al. 2018), including five to six mature individuals. Genetic studies, which only included material from the USVI, revealed relatively high levels of genetic diversity and found significant genetic differentiation between the two largest known localities at Nanny Point and Reef Bay (Stanford et al. 2013). Given these genetic studies and the recently found subpopulation on the island of Tortola, eight subpopulations have been identified.

#### Subpopulations

Abundance largest subpopulation: 108

Number of subpopulations: 8

Trend: Decline (observed)

Extreme fluctuations?: No

Severe fragmentation?: No

#### Habitat

System: Terrestrial

Habitat specialist: Yes

Habitat (narrative): This species' habitat is described as dry deciduous and coastal scrub forests with dry soils at lower elevations between 0 and 200 metres above sea level. This species usually grows as an understorey plant, responding favourably to disturbance (Lindsay et al. 2015, Heller et al. 2018).

Trend in extent, area or quality?: Decline (observed)

##### Habitat

Habitat importance: Major Importance

Habitats: 1.5. Forest - Subtropical/Tropical Dry

#### Habitat

Habitat importance: Major Importance

Habitats: 1.5. Forest - Subtropical/Tropical Dry

#### Ecology

Generation length (yr): 0

Dependency of single sp?: Unknown

Ecology and traits (narrative): The generation length of this species is unknown and further research is needed into the species ecology and reproduction.

#### Threats

**Figure 8.**
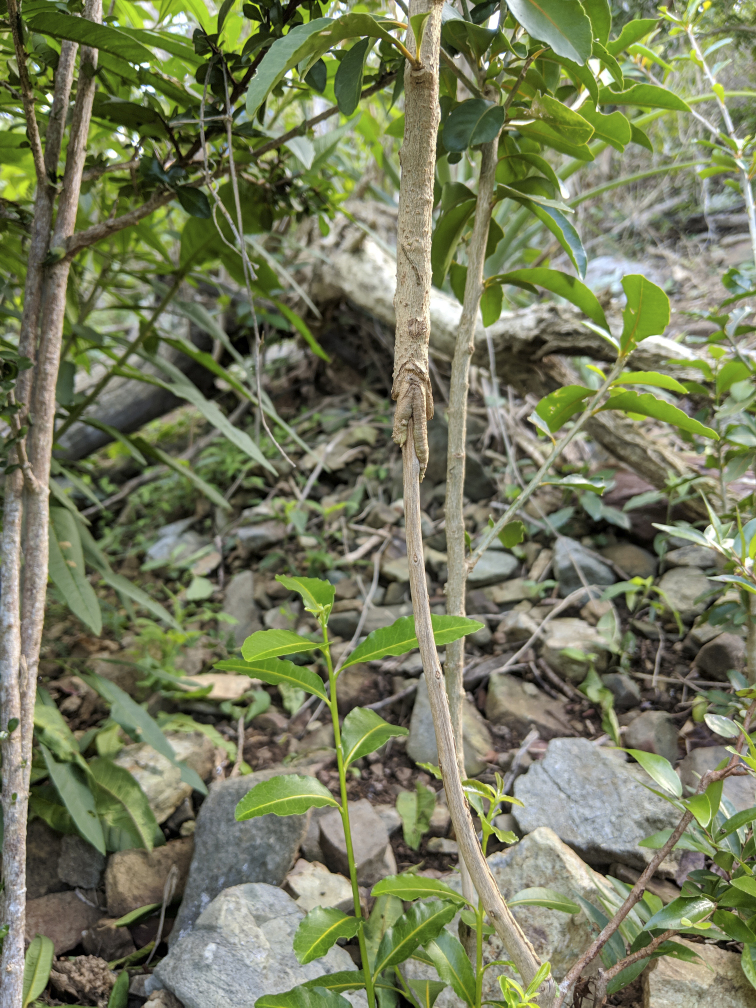
*Solanumconocarpum* stem damaged by feral animal grazing. Image by Thomas Heller.

Justification for threats: This species is subjected to several threats. Across its range, feral animals, goats on Tortola (BVI) and goats, pigs, donkeys and deer on the island of St. John (USVI) have been documented to graze on this species foliage and damage the bark of mature trees (Fig. 8) (Heller et al. 2018, Island Conservation 2018). These feral animals are further affecting the quality of the habitat, by altering soil conditions, as well as being vectors for invasive species. In addition, deer have been recorded targeting the fruits and, thus, are suspected to be the main cause for the lack of natural recruitment observed in the wild (Island Conservation 2018, U.S. Fish and Wildlife Service 2019). In the USVI, invasive scale insects, *Praelongortheziapraelonga* Douglas, 1891 and *Insignortheziainsignis* Browne, 1887, have been found attacking *S.conocarpum* at Nanny Point, while the invasive plants, *Megathyrsusmaximus* (Jacq.) B.K.Simons & S.W.L.Jacobs and *Leucaenaleucocephala* (Lam.) de Wit, have been reported to be encroaching on this species at the same location on St. John (U.S. Fish and Wildlife Service 2017, Island Conservation 2018, U.S. Fish and Wildlife Service 2019). Additionally, suitable habitat for *S.conocarpum* is being further fragmented because of urban development for residential and tourism infrastructure on Tortola (Heller et al. 2018). Catastrophic hurricanes associated with a changing climate are also suggested to be impacting this species' suitable habitat (Island Conservation 2018). In the BVI, it has been observed that the disturbance caused by the 2017 Hurricane Irma opened a pathway for the encroachment of invasive species, while on St. John, habitat modification has also been documented (Heller et al. 2018, U.S. Fish and Wildlife Service 2019). Individuals at Nanny Point in USVI may also be affected by sea surge associated with hurricanes and sea level rise due to their close proximity to the coast (U.S. Fish and Wildlife Service 2019).

##### Threats

Threat type: Ongoing

Threats: 1.1. Residential & commercial development - Housing & urban areas1.3. Residential & commercial development - Tourism & recreation areas8.1.2. Invasive and other problematic species, genes & diseases - Invasive non-native/alien species/diseases - Named species11.4. Climate change & severe weather - Storms & flooding

#### Threats

Threat type: Ongoing

Threats: 1.1. Residential & commercial development - Housing & urban areas1.3. Residential & commercial development - Tourism & recreation areas8.1.2. Invasive and other problematic species, genes & diseases - Invasive non-native/alien species/diseases - Named species11.4. Climate change & severe weather - Storms & flooding

#### Conservation

Justification for conservation actions: This species has been the focus of several conservation initiatives, mainly in the USVI. Most known individuals occur within the Virgin Islands National Park boundaries, on St. John. A habitat suitability model was developed for St. John aiming to identify undetected populations and to identify reintroduction sites (Palumbo et al. 2016). There are *ex-situ* collections established regionally on the islands of Tortola, St. Croix, St. John and Puerto Rico, as well as other botanic gardens in the USA (Hamilton et al. 2018, Heller et al. 2018, Island Conservation 2018). Seeds have also been banked at Kew's Millennium Seed Bank and DNA samples are stored in Kew’s DNA Bank. These *ex-situ* collections should be expanded to include material from both territories, from all subpopulations and from as many individuals as possible. Population genetics studies should equally be expanded to include more individuals and the newly discovered subpopulation (e.g. Tortola). There are ongoing efforts to propagate at least 100 individuals to enhance the known natural populations, assess the extent of invasive mammal species impacts and provide management recommendations for invasive mammals on the island of St. John (Island Conservation 2018, U.S. Fish and Wildlife Service 2019).

##### Conservation actions

Conservation action type: In Place

Conservation actions: 1.1. Land/water protection - Site/area protection3.3.1. Species management - Species re-introduction - Reintroduction3.4.1. Species management - Ex-situ conservation - Captive breeding/artificial propagation4.2. Education & awareness - Training4.3. Education & awareness - Awareness & communications

##### Conservation actions

Conservation action type: Needed

Conservation actions: 1.1. Land/water protection - Site/area protection3.4.2. Species management - Ex-situ conservation - Genome resource bank

#### Conservation actions

Conservation action type: In Place

Conservation actions: 1.1. Land/water protection - Site/area protection3.3.1. Species management - Species re-introduction - Reintroduction3.4.1. Species management - Ex-situ conservation - Captive breeding/artificial propagation4.2. Education & awareness - Training4.3. Education & awareness - Awareness & communications

#### Conservation actions

Conservation action type: Needed

Conservation actions: 1.1. Land/water protection - Site/area protection3.4.2. Species management - Ex-situ conservation - Genome resource bank

#### Other

##### Use and trade

##### Ecosystem services

#### Use and trade

#### Ecosystem services

#### Viability analysis

## Conclusion

Despite many decades of botanical exploration, knowledge gaps on the native flora of many of the UK Overseas Territories (UKOTS) remain (Clubbe et al. 2020), highlighting the need for frequent and continued botanical surveys. For more than twenty years, Kew’s UKOTs team and the National Parks Trust of the Virgin Islands (NPTVI) have been actively surveying the islands of the BVI Archipelago, consolidating botanical knowledge and assessing conservation needs. Over the past decade, Kew and NPTVI have also been working closely with regional partners, such as US Fish and Wildlife Service (USFWS), the Puerto Rico Department of Natural and Environmental Resources and the University of Puerto Rico to survey, study and conserve threatened and endemic plant species. Our collaborative and intensive survey efforts are now resulting in many new discoveries: new species records, range extensions and new plant records for the region. The discovery of *S.conocarpum* on Sabbath Hill on the island of Tortola is an example of the benefit of continued regional surveying efforts.

Strong regional collaboration is needed to conserve species such as *S.conocarpum* that cross international borders and prevent their extinction. Future conservation efforts should prioritise the exclusion or population control of feral animals within the species' habitat as this is the main threat to the viability of *S.conocarpum* populations. In addition, research on population genetics across the species range is needed to provide information for future propagation and reintroduction efforts. For the time being, this species is assessed as Endangered (EN), based on Criteria B1b(iii,v)+2b(iii,v)+C2a(i), according to the IUCN Red List Categories and Criteria (version 3.1) and guidelines (IUCN Standards and Petitions Committee 2019).

Conclusion

## Supplementary Material

8164F8D9-B32D-5E87-9210-CDC3ED7248A5 10.3897/BDJ.9.e69156.suppl1 Supplementary material 1 *Solanumconocarpum* occurrences Data type occurrences Brief description *Solanumconocarpum* occurrences. Data from Herbarium collections and human observations. File: oo_510464.kml https://binary.pensoft.net/file/510464 Barrios, S.
